# Short-term and intermediate outcomes of cardiogenic shock and cardiac arrest patients supported by venoarterial extracorporeal membrane oxygenation

**DOI:** 10.1186/s13019-021-01674-w

**Published:** 2021-10-09

**Authors:** Deep Vakil, Cassandra Soto, Zoee D’Costa, Lindsay Volk, Sivaveera Kandasamy, Deepa Iyer, Hirohisa Ikegami, Mark J. Russo, Leonard Y. Lee, Anthony Lemaire

**Affiliations:** Division of Cardiothoracic Surgery, Department of Surgery, RUTGERS-Robert Wood Johnson Medical School, 125 Paterson Street, New Brunswick, NJ 08903 USA

**Keywords:** Veno-arterial extracorporeal membrane oxygenation, Cardiogenic shock, Cardiac arrest

## Abstract

**Background:**

Cardiogenic shock and cardiac arrest are life-threatening emergencies with high mortality rates. Veno-arterial extracorporeal membrane oxygenation (VA ECMO) and extracorporeal cardiopulmonary resuscitation (e-CPR) provide viable options for life sustaining measures when medical therapy fails. The purpose of this study is to determine the utilization and outcomes of VA ECMO and eCPR in patients that require emergent cardiac support at a single academic center.

**Methods:**

A retrospective chart review of prospectively collected data was performed at an academic institution from January 1st, 2018 to June 30th, 2020. All consecutive patients who required VA ECMO were evaluated based on whether they underwent traditional VA ECMO or eCPR. The study variables include demographic data, duration on ECMO, length of stay, complications, and survival to discharge.

**Results:**

A total of 90 patients were placed on VA ECMO for cardiac support with 44.4% (40) of these patients undergoing eCPR secondary to cardiac arrest and emergent placement on ECMO. A majority of the patients were male (n = 64, 71.1%) and the mean age was 58.8 ± 15.8 years. 44.4% of patients were transferred from outside hospitals for a higher level of care and 37.8% of patients required another primary therapy such as an Impella or IABP. The most common complication experienced by patients was bleeding (n = 41, 45.6%), which occurred less often in eCPR (n = 29, 58% vs. n = 12, 30%). Other complications included infections (n = 11, 12.2%), limb ischemia (n = 13, 14.4%), acute kidney injury (n = 17, 18.9%), and cerebral vascular accident (n = 4, 4.4%). The length of stay was longer for patients on VA ECMO (32.1 ± 40.7 days vs. 17.7 ± 18.2 days). Mean time on ECMO was 8.1 ± 8.3 days. Survival to discharge was higher in VA ECMO patients (n = 23, 46% vs. n = 8, 20%).

**Conclusion:**

VA ECMO provided an effective rescue therapy in patients in acute cardiogenic shock with a survival greater than the expected ELSO guidelines of 40%. While the survival of eCPR was lower than expected, this may reflect the severity of patient’s condition and emphasizes the importance of careful patient selection and planning.

## Introduction

Despite recent advancements in medicine [1], cardiogenic shock (CS) and cardiac arrest remain life-threatening emergencies with high mortality rates [2, 3]. When conventional medical therapy fails, veno-arterial extracorporeal membrane oxygenation (VA-ECMO) and extracorporeal cardiopulmonary resuscitation (e-CPR) are possible life-saving options for patients who would otherwise succumb to cardiopulmonary failure [4–6]. In the setting of persistently compromised cardiovascular function, VA-ECMO provides hemodynamic support and delivers oxygenated blood to the body to maintain end-organ perfusion. In general, VA-ECMO is a bridge to myocardial recovery, bridge to decision making, or durable mechanical circulatory support (MCS) placement. The operative procedure can lead to complications and contribute to morbidity [7]. It is resource-intensive, requires a highly trained multidisciplinary team (MDT), and contributes to increased healthcare expenditures [8].

In recent years, VA-ECMO use for cardiopulmonary failure has increased [9] and therefore, it is crucial to evaluate the outcomes of VA-ECMO therapy. To be an effective therapy both from patient care and healthcare system perspectives, proper patient selection is paramount to minimize mortality, morbidity, and healthcare expenditures. In the absence of VA-ECMO clinical trials, cohort studies are crucial to elucidate new findings and contribute to meta-analyses. Our study assesses the clinical characteristics and outcomes of patients who underwent VA-ECMO for cardiogenic shock (E-CS) and cardiac arrest (E-CPR) at a large-volume center. The purpose of the study is to review the outcomes of a tertiary care center after initiating an ECMO program.

## Methods

This study is a retrospective chart review of 90 consecutive patients who were started on VA-ECMO for refractory cardiogenic shock (RCS) or cardiac arrest from January 1, 2018 to June 30, 2020. The patients either initially presented to or were transferred to an academic tertiary center and a level 1 trauma center. Study investigators compiled data pertaining to demographics, past medical history, transfer status, use of other MCS (ex. Impella or IABP), and outcomes. The primary outcome was survival to discharge. Secondary outcomes included duration on ECMO, length of stay (LOS), and complications. The complications examined included bleeding, acute kidney injury (AKI), limb ischemia, and cerebrovascular accident (CVA). Patient demographics, past medical history, type of ECMO cannulation (central or peripheral), and use of dual MCS therapy were also established. The study was approved by the institutional review board and ethics committee (NO: 2020000011).

### Procedure details

All the patients that were placed on VA ECMO had their cannulas placed either peripherally or centrally. The preference of the surgical team was for peripheral placement because this approach had several advantages including ease of insertion and removal of the cannulas. The placement of cannulas peripherally was done primarily by ultrasound guidance to identify the femoral artery and vein and then the seldinger technique was used to place the cannula with echocardiographic guidance. A distal perfusion catheter was placed in all patients who had peripheral VA ECMO placed. The cannula sizes varied based on the patient’s size and the flow required. Conversely, the cannulas used for central VA ECMO were identical to the cannulas used to go on cardiopulmonary bypass. In addition, the main indication for placement of central VA ECMO was post cardiotomy.

### Statistical analysis

Univariate and multivariable logistic regressions looking at survival as the dependent variable were conducted using R Version 3.6.3 (The R Foundation for Statistical Computing, Vienna, Austria). Complications (Bleeding, AKI, limb ischemia, infection, stroke, and other), demographics (age, male gender, BMI, and race), comorbidities (DM, HTN, HLD, CHF, COPD, ESRD/CKD, MI, CVA, and smoking), and ECMO factors (transfer, other primary therapy, ventilator support, AKI prior to ECMO, and cardiac vs. ECPR) were examined in separate models. A *p* value of 0.05 was used to determine statistical significance. Regression results are presented as odds ratio ± standard error.

## Results

**Fig. 1:**
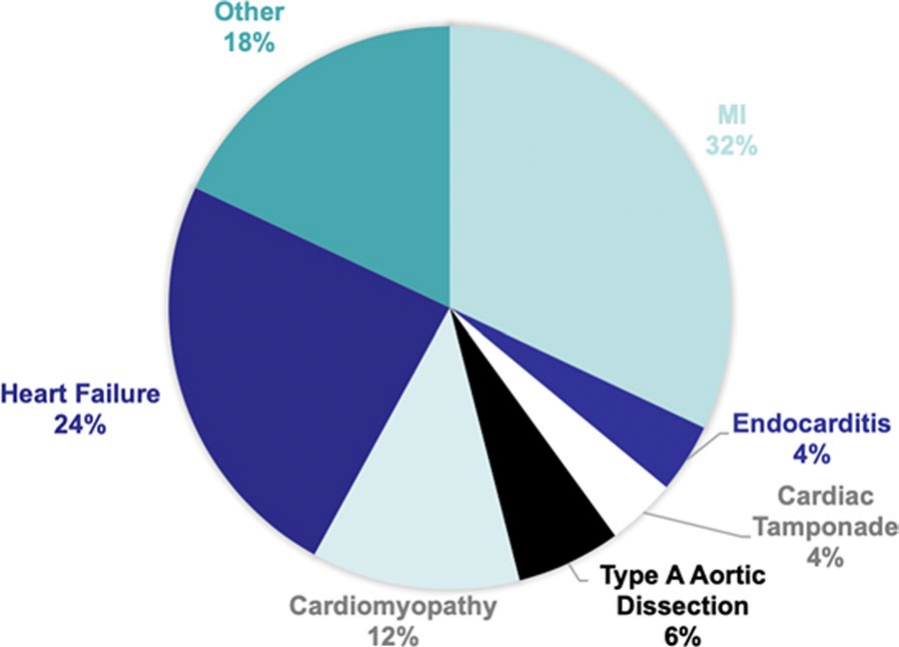
Causes of cardiogenic shock (*MI* myocardial infarction)

A total of 90 patients required VA-ECMO during the study period. Patients were primarily male (71%) and the mean age was 58.8 ± 15.8. Fifty patients (55.6%) required E-CS with a mean age of 60.0 ± 16.0 and 68% male. The causes of cardiogenic shock are demonstrated in Fig. 1. The most common causes of CS were myocardial infarction and heart failure. Forty patients (44.4%) required E-CPR with a mean age of 57.8 ± 15.6 and 75% male. Baseline patient characteristics are shown in Table 1. In the entire cohort of 90 patients, 44.4% were transferred from other institutions. The E-CS group had 30 (60%) transfer patients and the E-CPR group had 10 (25.0%). The E-CS group had 25 (50%) patients with dual MCS therapy and the e-CPR had 9 (22.5%). The E-CS and E-CPR groups had 80.0% and 92.5% patients with peripheral cannulation, respectively.

**Table 1:** Baseline demographic and clinical characteristics of E-CS and E-CPR patients

	All	E-CS	E-CPR
Patients, n (%)	90 (100)	50 (55.6)	40 (44.4)
Age (years), mean (SD)	58.8 (15.8)	60.0 (16.0)	57.4 (15.6)
Gender			
Male, n (%)	64 (71.1)	34 (68.0)	30 (75.0)
Female, n (%)	26 (28.9)	16 (32.0)	10 (25.0)
BMI, mean (SD)	30.7 (6.96)	29.1 (7.28)	32.9 (5.93)
Race			
White, n (%)	43 (47.8)	24 (48.0)	19 (47.5)
Black, n (%)	16 (17.8)	9 (18.0)	7 (17.5)
Asian, n (%)	13 (14.4)	8 (16.0)	5 (12.5)
Other, n (%)	18 (20.0)	9 (18.0)	9 (22.5)
Transfers, n (%)	40 (44.4)	30 (60.0)	10 (25.0)
Dual MCS therapy (Impella, IABP)	34 (37.8)	25 (50.0)	9 (22.5)
Diabetes, n (%)	33 (36.7)	17 (34.0)	16 (40.0)
Hypertension, n (%)	59 (65.6)	31 (62.0)	28 (70.0)
Hyperlipidemia, n (%)	39 (43.3)	23 (46.0)	16 (40.0)
Congestive heart failure, n (%)	20 (22.2)	14 (28.0)	6 (15.0)
Peripheral vascular disease, n (%)	8 (8.9)	6 (12.0)	2 (5.0)
Coronary artery disease, n (%)	56 (62.2)	31 (62.0)	25 (62.5)
Chronic obstructive pulmonary disease, n (%)	7 (7.8)	4 (8.0)	3 (7.5)
CKD/ESRD, n (%)	12 (13.3)	9 (18.0)	3 (7.5)
Past myocardial infarction, n (%)	21 (23.3)	11 (22.0)	10 (25.0)
Past cerebrovascular accident, n (%)	10 (11.1)	8 (16.0)	2 (5.0)
Smoking, n (%)	22 (24.4)	11 (22.0)	11 (27.5)
Ventilator support, n (%)	69 (76.7)	35 (70.0)	34 (85.0)
AKI/ RF (prior to ECMO initiation), n (%)	12 (13.3)	10 (20.0)	2 (5.0)

*AKI* acute kidney injury, *BMI* body mass index, *CKD* chronic kidney disease, *ECMO* extracorporeal membrane oxygenation, *E-CPR* extracorporeal cardiopulmonary resuscitation, *E-CS* venoarterial extracorporeal membrane oxygenation for cardiogenic shock, *ESRD* end-stage renal disease, *IABP* intra-aortic balloon pump, *MCS* mechanical circulatory support, *RF* renal failure, *SD* standard deviation

Overall, 34.4% of the patients survived to discharge. The survival to discharge rates for E-CS and E-CPR were, respectively, 46.0% and 20.0%.

**Fig. 2:**
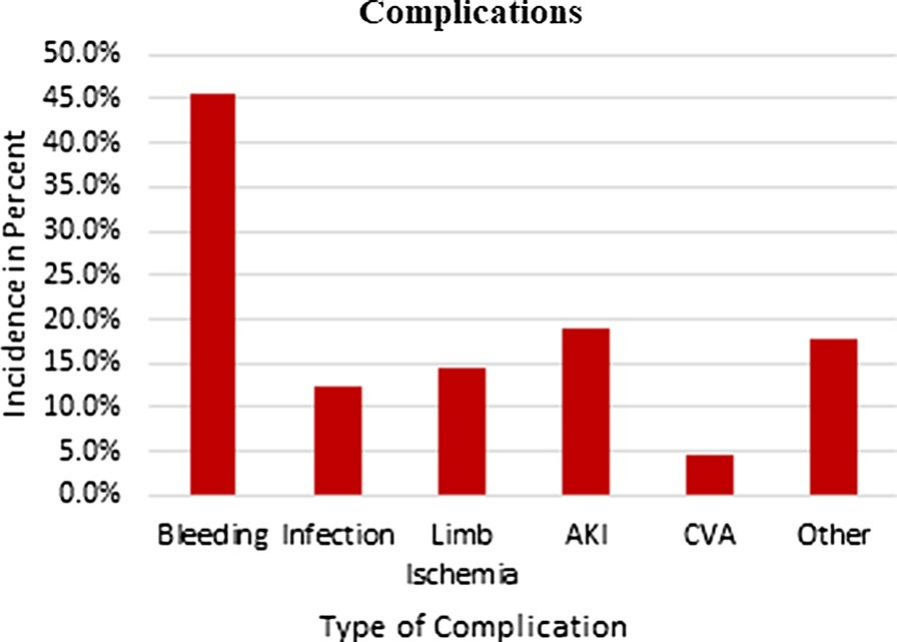
Complications of all patient requiring venoarterial extracorporeal membrane oxygenation (*AKI* acute kidney injury, *CVA* cerebral vascular accident)

Table 2 demonstrates patient outcomes. Seventy percent of all the patients placed on VA-ECMO had complications. Overall, the most common complications were bleeding (45.6%) and AKI (18.9%). Figure 2 displays the complications for all the patients placed on VA-ECMO. The complications for the E-CS group from most to least frequent: bleeding (58.0%), AKI (22.0%), infection (16.0%), limb ischemia (12.0%), and CVA (6.0%). The complications for the E-CPR group included bleeding (30%), limb ischemia (17.5%), AKI (15.0%), infection (7.5%), other (5.0%), and CVA (2.5%). The E-CS cohort had a mean LOS of 32.1 ± 40.7 days with a mean ECMO duration of 8.7 ± 8.3 days. The E-CPR cohort had a mean LOS of 17.7 ± 18.2 days and a mean ECMO duration of 7.2 ± 8.5 days.

**Table 2:** Patient outcomes

	All	E-CS	E-CPR
Duration on ECMO, mean days (SD)	8.1 (8.3)	8.7 (8.3)	7.2 (8.5)
Length of stay, mean days (SD)	25.7 (33.3)	32.1 (40.7)	17.7 (18.2)
Complications			
Bleeding, n (%)	41 (45.6)	29 (58.0)	12 (30.0)
Acute kidney injury, n (%)	17 (18.9)	11 (22.0)	6 (15.0)
Limb ischemia, n (%)	13 (14.4)	6 (12.0)	7 (17.5)
Infection, n (%)	11 (12.2)	8 (16.0)	3 (7.5)
Cerebral vascular accident, n (%)	4 (4.4)	3 (6.0)	1 (2.5)
Other, n (%)	16 (17.8)	14 (28.0)	2 (5.0)
Survival to discharge, n (%)	31 (34.4)	23 (46.0)	8 (20.0)

*ECMO* extracorporeal membrane oxygenation, *E-CPR* extracorporeal cardiopulmonary resuscitation, *E-CS* venoarterial extracorporeal membrane oxygenation for cardiogenic shock, *SD* standard deviation

Presence of infection was a positive predictor of survival in both the univariate and multivariable model (univariate: 1.389 ± 0.673, *p* = 0.039, multivariable 2.051 ± 0.875, *p* = 0.019).

Increasing BMI was associated with decreased survival in both the univariate and multivariate models (univariate: − 0.090 ± 0.038, *p* = 0.017, multivariate − 0.104 ± 0.040, *p* = 0.0095). Increasing age was also associated with decreases survival, but only in the multivariate model (− 0.037 ± 0.017, *p* = 0.035). None of the comorbidities examined were statistically significantly associated with survival in either the univariate or multivariable analyses. In addition, both ventilator support (univariate: − 1.811 ± 0.541, *p* = 0.0008, multivariable: − 2.114 ± 0.650, *p* = 0.0011) and ECPR (univariate: − 1.226 ± 0.487, *p* = 0.012, multivariable: − 1.649 ± 0.604, *p* = 0.006) were negatively associated with survival. The presence of AKI or renal failure prior to ECMO initiation was negatively associated with survival in the multivariable model (− 2.454 ± 1.064, *p* = 0.021).

## Discussion

The data from our study shows that the VA ECMO experience was successful compared to national results. A total of 90 consecutive VA-ECMO patients (50 for E-CS and 40 for E-CPR) during the study time period were evaluated. In the E-CS cohort, the survival to discharge was 46.0% which is higher than the reported rates of 24.4 to 42.0% [4, 10–18] and 44% from the Extracorporeal Life Support Organization (ELSO) Registry [19]. The E-CPR cohort survival to discharge rate was 20.0%, which is within the reported survival rates ranging from 13.6 to 34.1% [5, 6, 11], however, lower than ELSO’s 29% [19]. The findings are very comparable to the various results found in the literature.

The success of an ECMO program depends on accurate patient selection. Patients with reversible underlying pathophysiology are most likely to benefit. It is critical to consider if patients may be too high risk to benefit from ECMO. Smith et al. found that there was a significantly higher mortality rate among patients who had VA-ECMO for 4 or fewer days because a large number of these early case fatalities had either organ failure or a diagnosis incompatible with life [20]. The finding suggests that by the time ECMO was initiated the damage from tissue hypoperfusion was severe and irreversible. In our study, the average duration on ECMO for E-CS was greater than that reported by Smith et al. [20] Careful patient selection likely prevented the initiation of ECMO in patients that were too ill to benefit. As a result, our cohort had fewer patients who were discontinued from ECMO early on and thus leading to a higher average ECMO duration. This could be a reason why our E-CS survival rate was higher than that reported by both Smith et al. and ELSO [19, 20]. Plausibly, the E-CPR cohort had shorter ECMO duration early on because they had severe and irreversible organ damage. This could have contributed to our E-CPR survival rate being lower than that reported by ELSO [19]. As a result, although a patient may have a reversible cause of cardiogenic shock, if the resulting end-organ damage is severe, irreversible, and incompatible with life, salvage attempts may not be possible.

Other important aspects of patient selection are age and comorbidities. Increasing age and patient comorbidities are associated with mortality [7, 11–14, 16, 21, 22]. Kaushal et al. reported a survival rate of 40.8% at an academic quaternary care center for which average age was 53.5 ± 15.3 and had the following comorbidities (%): hypertension (HTN) (57.87), diabetes mellitus (DM) (34.89), peripheral vascular disease (4.26), hyperlipidemia (HLD) (28.51), coronary artery disease (CAD) (28.09) [11]. A study of VA-ECMO in CS patients by Truby et al. reported their survival to discharge rate of 38.6% with the following patient characteristics: mean age 56.85 ± 16.10, CAD (45.8), HLD (40.2), HTN (57.5), chronic obstructive pulmonary disease (8.8), DM (29.1), and prior CVA (8.4) [14]. Despite having a higher average age (60 ± 16.0) and a higher frequency of comorbidities, our institution’s E-CS cohort had a higher survival rate. This suggests our institution’s higher E-CS survival rate in part stems not only from identifying which patients will most benefit from ECMO, but also the appropriate management of the patients throughout their hospital course.

The hospital course of VA-ECMO patients can be tested by complications, related to the underlying disease process, ECMO apparatus, or both. Compared to a meta-analysis of complications in E-CS and E-CPR by Cheng et al. [23], the current study’s E-CS cohort had a higher bleeding complication rate. This could in part be due to our cohort’s higher average duration on VA-ECMO, rendering a longer duration of anticoagulation therapy than that of the majority of the studies in the meta-analysis. However, for other complications, our experience was within or less than the reported rates. The entire VA-ECMO cohort and the sub-cohorts, E-CS and E-CPR, had AKI and infection rates that were less than those in the meta-analysis. Our academic tertiary institution utilizes multidisciplinary resources to manage the challenging and dynamic hospital course of patients with cardiac failure and could be a reason for the lower complication rates. This is supported by Dalia et al. who found that the additional support including cardiac anesthesia specialists, intensivists, cardiology heart failure specialists, and other specialist improves outcomes [24].

Management and prevention of these complications are crucial. A recent study by Kaushal et al. found that limb ischemia is an independent predictor of mortality [11]. Our E-CS cohort had a limb ischemia rate of 12.0%, which is lower than that reported by Cheng et al. [23] and is consistent with our study’s higher E-CS survival rate compared to other reported survival rates. Reduced 24-h urine output and prothrombin activity of less than 50%, which are early signs suggestive of kidney and liver failure, respectively, are associated with mortality [4, 22, 25]. These are the initial steps of a dangerous sequela that concludes with multiorgan failure, a common cause of mortality in this patient population [4, 11, 12]. These findings highlight the importance of early identification of complications which can allow for earlier intervention and lower mortality and morbidity. More research is needed on evaluating factors that are associated with a higher risk of developing complications. This information can be used by an interdisciplinary team to stratify the risk of complications and cautiously monitor or evaluate patients at higher risk. Involving a collaborative team of specialties including vascular surgery, cardiology, and nephrology can ensure preparation to intervene before severe and irreversible damage that is incompatible with life. This knowledge can also be utilized in the training of intensive care unit providers to improve surveillance and prevent complications.

## Limitations

As a retrospective analysis which has limitations inherent to retrospective studies. Information such as medical therapy, ejection fraction, and cardiac index before VA-ECMO initiation were not evaluated. Time to cannulation and duration of CPR prior ECMO initiation were not evaluated. This was an observational study at a single center without any control or randomization.

## Conclusion

E-CS is an effective rescue therapy with a survival greater than the expected ELSO guidelines of 44%. While the survival of E-CPR was lower than expected, this reflects the severity of the patient’s condition and it emphasizes the importance of careful patient selection. This study provides evidence for the utility of VA-ECMO as salvage therapy in patients with cardiac compromise who otherwise would have fatal outcomes. Further research must be done to improve patient selection. Research to improve the prevention and early identification of complications is also required. New findings in these areas will further improve the mortality, morbidity, and healthcare expenditure associated with cardiogenic shock and cardiac arrest.

## Data Availability

Available upon request.
